# From clicks to connection: designing nursing content within an electronic health record that reflects person-centred relationships and choices

**DOI:** 10.3389/frhs.2026.1781450

**Published:** 2026-03-25

**Authors:** Michele Hardiman, Judith Watkin, Winifred O'Neill, Sinead Hanley

**Affiliations:** 1 Blackrock Health, Galway Clinic, Galway, Ireland; 2 Centre for Person-centred Practice Research, Queen Margaret University, Edinburgh, United Kingdom; 3 Department of Nursing and Health Science Castlebar, Atlantic Technological University, Castlebar, Ireland

**Keywords:** documentation (nursing), electronic health record, nursing, person-centredness, technology, organisational culture

## Abstract

Electronic Health Records (EHRs) are central to healthcare transformation, promising improved access to information, decision support, and safer care delivery. It acts as a central repository of all information pertinent to a person receiving treatment and is easily accessible. This paper presents a an experiential perspective and follow up from a nursing team who led the design, development, and evaluation of a person-centred electronic nursing record within an acute hospital setting. Guided by the Person-centred Practice Framework and participatory approaches to workplace culture development, the team collaborated with informatics specialists to redesign more than 120 assessments, diagnoses, interventions and outcomes that better reflect nursing values, patient choice, and local workflows. Through continuous attention to organisational structures, facilitative processes, and emerging cultural patterns, nurses achieved greater alignment between digital documentation and the realities of bedside practice. Evaluation data demonstrated improved nurse engagement with the EHR, enhanced therapeutic relationships, reduced documentation burden, and strengthened person-centred cultures across the organisation. These findings highlight the critical role of nurses in digital transformation and the need for meaningful input into EHR content design and governance. We argue that digital nursing documentation must reflect the complexity and relational nature of nursing practice, rather than prioritising technical tasks or disease-focused content. As national and international EHR initiatives advance, nursing leadership must influence system procurement, content customisation, and ongoing evaluation to ensure that digital records uphold professional values and support compassionate, holistic, person-centred care.

## 1 Introduction

There is much debate emerging in healthcare literature related to Electronic Health Records (EHR's) both positively and negatively. The global debate about digital records and advantages/ disadvantages is largely focused on technology use and decision supports available, rather than a focus on content and specifically the content in capturing nursing interventions (1). In most cases, nursing content is presented in a preset format supported by automated clinical support functionality. The nurse reviews this preset content and ‘ticks’ to say what care has been delivered. This should make audits and monitoring adherence to clinical standards a relatively simple task. However, we believe this perspective oversimplifies nursing care pathways and fails to capture the complexity and multifaceted nature of the nurse–patient relationship. In this paper, the authors wish to offer an alternative view, exploring the possibilities of using the nursing component of an EHR to capture and support the relationship between the nurse and the person receiving care. We would also like to share our experience of how redesigning the nursing components of an existing EHR to better reflect our person-centred culture, has influenced both organisational culture and the quality of care in an acute hospital setting. The authors acknowledge that changing systems alone is insufficient in achieving a person-centred culture and must go hand in hand with purposeful facilitated attention to the systems, processes and patterns that result in person-centred workplaces and care environments.

## 2 Background

This paper is presented as an experiential perspective offering shared learning and insights from a nursing team that has designed and implemented a person centred nursing record within an electronic health record (EHR). It builds on a previously published account detailing the development of a nursing record aimed at supporting compassionate, person centred nursing practice (2).

The work is situated within a mid-sized private acute general hospital in the Republic of Ireland, which has been recognised as a national leader in digital innovation since 2008, when it became the first hospital in Ireland to implement an integrated EHR. The organisation further advanced its digital maturity by achieving the HIMSS Stage 6 designation in 2014, an award granted to healthcare institutions that demonstrate a high level of integration and utilisation of digital clinical systems. In 2015, the hospital planned a major upgrade with the existing vendor to go-live in 2017. The nursing team had previously established values and principles for practice underpinned by the Person- Centred Practice Framework (30), incorporating principles of collaboration through inclusion and participation as the key to a person-centred culture and delivering person-centred care.

Leveraging this upgrade as a catalyst for transformation, the nursing team collaboratively developed a person centred nursing record aligned with established nursing workflows and underpinned by national and international evidence-based practice guidelines. This process spanned a two-year period supported by nursing leaders who had been also part of a participatory action research doctoral study focused on facilitating person-centred cultures. Their additional knowledge and understanding of person-centredness enhancing their focus while designing the EHR. Nursing content within an EHR is traditionally driven by pre-set functionality with many organisations using standardised nursing language. The team developed more than 120 new documents, including assessments, nursing diagnoses, and person-centred interventions (representing care plans) and their associated evaluations. These were informed by evidence-based protocols, NANDA-I diagnoses (3), NIC interventions (4), and NOC outcomes (5), with particular attention to supporting patient choice and honouring individual values and beliefs. Although the work was complex, the result is a nursing record that mirrors the natural flow of practice, making it intuitive and easy to use at the point of care.

Following the system's go-live in 2017, a research programme was initiated in 2019 using a participatory methodology and supported by the Person-Centred Research Centre at Queen Margaret University, Edinburgh. The purpose of this programme was to evaluate how the Complete Electronic Nursing Record guided by the Person-centred Framework (30) influenced person-centredness within the hospital setting. A range of evidence-based evaluation tools were used to gather data, including the Person-centred Practice Inventory (Staff) (6), the Workplace Critical Culture Analysis Tool (WCCAT) (7), Person-centred Moments,^1^ patient interviews, and co-researcher reflections. These data sets were thematically analysed using Braun and Clarke's (8) approach to explore individuals' experiences of person-centredness within the hospital.

Although this work was not formally published, the findings indicated a strong relationship between nurses' bedside presence, the recording of personalised information (such as preferred name, what matters to the person, and individual care preferences), and a positive care experience for both patients and staff, characterised by a sense of involvement in care (30). These evaluation tools have been repeated at intervals as part of the person-centred programme in the hospital to maintain focus on sustaining person-centred cultures.

## 3 Capturing person-centred care

Keeping person-centeredness to the forefront of emerging digital healthcare is central to nursing, medicine and allied health care guidance and policy in many countries (9–11), however little attention has been applied to capturing person-centred interactions in digital format. In contrast, we conclude that person-centred content can be designed and included within the EHR to capture the essence of the relationship between the patient and their nurse whilst also meeting the standards and requirements for nursing documentation.

McCance and McCormack (12) suggests that person-centred practice requires more than the articulation of values underpinning therapeutic relationships between the persons delivering and receiving care. Instead, person-centred practice emerges when all relationships, systems, policies, and practice are underpinned by shared values; respect, mutual trust and understanding. Delivering care that supports empowerment, shared decision making and personal choice is no longer an optional add-on to technical and clinical care when a person is most notably at their most vulnerable to losing their personhood [(13) p.171]. The largest group of clinical users in a hospital setting is nursing. Despite this, in most cases, nurses have had minimal input in the content design, system usability, and adaptability at organisational investment level, prior to purchase or at design stage. Also, to date, EHR Vendors have not engaged in understanding nursing workflows and how that impacts on the useability of the EHR. In its place, they offer standardised content largely focused on disease management and medicalised interventions (14). This may in part account for the findings by Forde-Johnson et al. (1), whose systematic review identified overall nursing dis-satisfaction with the quality of electronic documentation. The authors concluding that nursing EHR content does not respect nursing workflows and is driven mainly by risk assessments and task focused interventions. As an outcome, the records primarily capture technical interventions and medical treatments with less content capturing the relationship between the nurse and the person, and the care delivered.

McCormack (15) suggests, we should be cautious in the rush to move to electronic records powered by preset algorithms or artificial intelligence (AI) and suggests it adds to the burden on nurses and fails to focus on the humanistic needs of the person as a unique individual. We concur that the fast-approaching use of AI in digital health may pose some additional challenges if it goes unchecked or takes away clinical human judgement, collaboration with the patient and with the multidisciplinary team. However, preset algorithms may offer evidence-based prompts to a nurse to support collaborative decision making.

## 4 Recording care

Over the last few decades, the specific documentation that nurses, especially Registered Nurses are expected to complete has been increasing across the western world. Documentation is often promoted as being a communication resource and a means for enhancing patient safety (16–18). Bøgeskov and Grimshaw-Aagaard (19), found that nurses were divided between a positive view of documentation as something essential, and a negative one of it being a meaningless burden that distracts nurses from their “real” work, contradicts their professional identity, and does not benefit patients. If findings such as those described remain unresolved the positive impact of an EHR may be negated by lack of trust by the user or refusal to engage with the process.

Given the amount and complexity of documentation, now said to be necessary for effective care, electronic records systems are claimed to offer better collection, storage, and retrieval of information (11). An EHR should empower, support, and improve the patient's experience of healthcare environments and enable staff to build more personalised plans of care (10). If supported, the provision of sufficient hardware to enable bedside recording of care will increase the opportunity to be present with a patient for longer (20). The underlying philosophy of person-centred practice can be compromised by the introduction of an EHR if its content is not carefully reviewed and adapted to align with local culture and values (2).

Whilst EHR's undoubtedly benefit many healthcare practitioners, primarily through improved access to patient information, benefits are enabled or hindered by material and social interests that exist within the workplace and organisational cultures (19, 21) which ultimately have a significant effect on acceptance and use of EHRs by the end user. Thus, an EHR needs both utility and meaning in regard to material and social practices to be successfully implemented and adopted by the end user. To achieve this, we use a Community of Practice approach (31), where work-based facilitators feedback and feedforward reflections on practice, documentation and culture focusing on what is working and what needs to be changed.

It has been questioned if an EHR is suitable for nursing documentation at all, particularly in relation to challenges to face-to-face communication (1). We would argue that it is appropriate, if the design, content, and process is owned by the end user, is aligned with local culture and nursing workflows. However, even where these conditions are in place nurses require further education and support to document patient care according to the nursing process, using international terminology to increase patient safety and improve the quality of documentation itself. Refocusing on the nurturing of a person-centred culture and practice and acknowledging un-intentional use of discriminatory or paternalistic language in our narrative notes is also necessary (22). Attention to use of person-centred language and customising content prior to the introduction of the nursing EHR may also help to promote and record person-centred relationships between the nurse and the patient (11).

## 5 Addressing structures, processes, and patterns

McCormack et al. [(23), p. 19] propose that healthcare organisations consist of interconnected systems made up of three key elements: Structures, such as purpose, vision, roles and responsibilities; Processes, which describe how these structures are put into action, including patient pathways, funding and purchasing; and Patterns, which reflect the organisation's culture. The authors, citing Plesk (24), suggest it is the integration of each of the three elements that enable change to occur. Paying attention to cultural patterns that are less tangible or easily measured, they play a crucial role in shaping organisational effectiveness.

Table 1 presents the structures, processes, and emerging patterns that have become evident since the organisation prioritised the development of a person-centred culture, supported and reinforced through documentation within the EHR.

**Table 1:** Systems elements: structures, processes, patterns [adapted from McCormack et al. (23), p. 28 organisational systems in developing a person-centred EHR].

Structures	Processes	Patterns
Organisation goals and strategy.	Patient journeys, care pathways.	Satisfaction with nursing EHR by bedside nurses.
Investment in EHR infrastructure (hardware and software).	Supporting and facilitated person-centred cultures.	Happier more contented staff.
	Shared values and vision Community of Practice—Work based facilitators.	Reduction of incidents and accidents.
Roles and responsibilities to ensure alignment with organisational values.		
		Effective person-centred relationships.
	Informatics and IT Support to redesign content.	
		Whole system approach.
	EHR Content Governance Interconnective processes such as nursing, pharmacy, medicine, AHP, laboratory, OR.	Holistic care.
	Collaboration, inclusion, and participation of all stakeholders	

## 6 Structures

The decision to introduce an EHR is a very costly and complex process, requiring macro-organisation support whose focus is largely on the system and vendor choice, implementation and ongoing support costs and benefits.

The off-the-shelf content available to nurses is, by design, prescriptive and primarily centred on medicalised, disease-focused issues presented within standardised nursing language frameworks. A key challenge lies in adapting internationally developed standardised nursing content so that it aligns with national, organisational, and cultural contexts. Our experience showed that gaining a deep understanding of system-level constraints and functionality, while also adhering to national standards and protocols grounded in agreed person-centred principles, provides essential direction for the Standardisation process.

Nursing informatics professionals, who combine IT qualifications with a nursing background are essential to facilitate the technical changes required to customise EHR content and processes. These professionals must work collaboratively with nursing management, bedside nurses, and the vendor to ensure success. A key question to be posed during the design and build phase of the implementation is: Should the clinical workflow be altered, or should the EHR process be customised/adapted?' Evidence-based workflows should guide the response. Stanhope and Matthews (25), suggest that currently EHR's are formatted to focus on the biological person and constrains the assessment of the individual as a holistic, unique, and complex being. Stanhope and Matthews (25), study found incorporating person-centred content into systems that were “locked in” during technical implementation stage resulted in less than satisfactory outcomes and adoption of workarounds to meet the needs of the nurses.

### 6.1 Tensions and tradeoffs in person-centred EHR design

Introducing an Electronic Health Record (EHR) system represents a significant organisational change, one that is inherently disruptive, complex, and challenges established norms (26). As our project expands to include two additional hospitals within the group, we face increasing pressure to justify the layout, content, and overall value of our person-centred approach. A critical aspect of this justification involves collecting and presenting data that demonstrates improvements in clinical standards, such as infection rates, hospital-acquired pressure injuries, and patient feedback. These indicators help illustrate the connection between person-centred practices, meaningful bedside relationships, and the way care is recorded in the EHR.

Planning for and supporting change is essential to reduce resistance and achieve quality outcomes that meet both organisational and nursing needs. This is particularly relevant for nurses, who are continuously introduced to new procedures and equipment and are expected to adapt quickly and seamlessly. While negotiations and tradeoffs are inevitable, drawing from past experience, maintaining open dialogue, and collaborating closely with the team help ground expectations and strengthen engagement. The presence of a dedicated gatekeeper who understands the system's functional capabilities and can facilitate agreed principles and values helps us minimise conflict and highlight system benefits. We believe that much of our success can also be attributed to the strong support and commitment from our organisation's leadership. This enabled the team to establish and sustain clear communication channels with bedside nurses and to foster a shared commitment to the values and vision underpinning the EHR.

## 7 Processes

Having a process to develop meaningful content that will harness the benefits of a digitalised record while also capturing (in so far as is possible in any format) the relationship, values, preferences, and choices for the patient is essential (2). The introduction of a person-centred EHR stands on a platform of person-centred practice and a focus on facilitation of a culture that enables others. The Person-centred Practice Framework (12), facilitated through clinical staff who are work-based facilitators, offered a pathway to support a collaborative process of introducing, supporting, and sustaining a practice culture that delivered on person-centredness within the hospital systems and processes, including within the EHR. This purposeful activity is central to maintaining a person-centred culture and is also a time for teams to reflect on current practice, patient safety, and culture issues (27, 28).

Forde-Johnson et al. (1), suggest that efforts to provide and document care to the standards required, can create a perceived shift away from the bedside and potentially exacerbated a cognitive dissonance, switched off thinking or ambivalence to the EHR. In contrast, we propose that creating the conditions where nurses engage in critical discussion and feel empowered, is needed to support professionalism and critical thinking. Challenging the system and critical analysis of usability to eliminate unnecessary tick boxes promoting critical thinking and professional practice can reduce the burden and frustrations expressed by nurses (14).

## 8 Focus on design

What EHR vendors offer, is a technical solution that integrates all of the moving parts of a highly intelligent Information system that collects extensive “data” about illness and treatments in a one size fits all presentation for hospitals. We suggest this is where nurses become passive recipients of the system functionality as it often presented as unalterable, un-customizable without excessive and unnecessary (according to vendor) cost. Collaborative working with informatics and IT colleagues who also work toward person-centred principles to achieve improved outcomes for patients and satisfaction from staff is essential. There is a universal agreement internationally that nurses need to have greater input into re-design and on-going governance of the nursing content in the EHR (1, 25, 29). Where this is achieved and actioned, we propose the resulting adoption of the nursing record is smooth and unchallenged.

## 9 Emerging and evolving patterns

Patterns reflect the true influence of organisational culture and what emerges when systems and processes are aligned with a shared vision. They reveal outcomes, intended or unintended, positive or negative, often linked to the interactions and relationships among all individuals [(23), p. 23]. After more than 20 years of working with an EHR, we can now see the benefits of having a comprehensive repository of all information relating to each patient. However, the EHR represents only one component in developing systems and processes that enhance care. It also enables us to identify patterns in patient care, treatment anomalies, relational dynamics, and cultural influences. In addition, the system generates data that supports audit, clinical standards, patient safety, cost management, and overall efficiency.

Our lived experience of using an integrated EHR in an acute hospital has evolved over time from general acceptance of the limitations in vendor content to finding workarounds through local manipulation by the IT Nurses. We can identify with the frustrations in working with and around ineffective design as described by Staggers et al. (14),. Satisfaction with EHR nursing content and workflows by bedside nurses when redesigning during a system upgrade was of vital importance.

Including the voices of bedside nurses in developing the EHR content has not only led to a more comprehensive and improved record, but has also empowered the nursing team by helping them feel valued and included. This approach supports and reinforces an ongoing person-centred culture [ (12) p. 25]. We continue to reflect on our practice, considering what we do well and where improvements can be made. Regularly reviewing the EHR, observing nursing workflows, and recognising patient preferences and choices in daily interactions helps us remain focused on person-centred care. Embedding this culture across all education sessions, workshops, and meetings has contributed to unintended yet welcome outcomes, such as a steady reduction in incidents, accidents, and complaints. The consistent presence of nurses at the bedside has also increased their visibility and strengthened patient engagement.

Since developing the EHR with person-centred options and supports, we have found that it promotes a respectful, person-centred environment for both patients and staff. Recent staff surveys indicate that nurses are satisfied with the EHR content and do not view documentation as a burden. We believe this positive environment has contributed to low staff vacancy rates and the recruitment of highly experienced nurses.

## 10 Discussion

Introducing a nursing EHR is a complex process, not just because it implies new and therefore extra work, it requires the introduction of new routines in core care delivery and introduces new performance requirements around recording in the EHR. Documentation contributes significantly to nursing workload. More than this it has become an ever-increasing requirement to support practice and provide evidence of care. It can be described as a burden or an essential component of care delivery and part of the nursing process.

There is an urgent need for nurses to be directly involved in software design to ensure that the essence and complexity of nursing is not lost in the system. An EHR should reflect the flow and practice of the nurse, the relationship between the nurse and the patient and the culture of care in the context where care is delivered. Unfortunately, most do not, because there is an over-focus on the system rather than the process.

Utility alone is not enough. The (positive) meaning in documentation is achieved by the nurses relating documentation to the ethical values of their profession, in this case, helping someone. It is important therefore in the development of an effective nursing EHR that the organisation has a strategy and the capability to challenge the software and customise the content. To achieve positive outcomes for the patient record and for the nurse usability, investment in content design to fit with local, national and international standards and goals is essential. This will require investment both locally and nationally to agree on the core element of a nursing record that flows in line with the relationship between the nurse and the patient and the workflow of the practice setting. Whilst the system may be intuitive, its principles, language, and priorities must be carefully structured and embedded within the EHR to support these goals.

## 11 Conclusion

The widespread adoption of electronic health records (EHRs) across all healthcare settings is inevitable and represents a meaningful advancement for patient safety and documentation accuracy. At the same time, addressing usability challenges experienced by end-users must remain an urgent priority (14). Nursing documentation varies significantly worldwide, reflecting differences in nursing roles and scopes of practice. Yet, despite this diversity, the fundamental purpose of nursing, providing compassionate, holistic, person-centred care, remains consistent. For that reason, the assessment, diagnosis, and delivery of care should be documented with the same level of importance as medical treatment within the patient record.

As nurses, we believe it is essential to reach consensus on the content and structure of digital nursing documentation. We also advocate moving away from fragmented or inconsistent digital record formats, toward a standardised national framework that clearly defines nursing pathways and content. With national EHR systems currently being procured, it is crucial that nursing leadership plays a central role in vendor selection, ensuring that usability for nurses, who form the largest user group in acute care, is prioritised. We encourage international nursing colleagues to engage in discussion and debate to help shape systems that truly meet the needs of the profession. Continued research is urgently needed to broaden and deepen the conversation on what nursing content should be captured within the EHR.

## Data Availability

The original contributions presented in the study are included in the article/Supplementary Material, further inquiries can be directed to the corresponding authors.

## Footnotes

1 Dewing J, The Donegal Project Team. *Person-centred Moments: Record Sheet (Version 1)*. Unpublished tool. (2017). Available online at: http://www.pcp-icop.org/resources
